# Prevalence of zoonotic nematode *Calodium hepaticum* varies with small mammal community diversity across a heterogenous landscape in Eastern Uganda

**DOI:** 10.1017/S0031182026101723

**Published:** 2026-04

**Authors:** Emilia Johnson, Diana Ajambo, Maria Capstick, Moses Arinaitwe, Olivia Ericsson, Fred Besigye, Jayna Raghwani, Tristan P W Dennis, Ronald Bogere, Andrina Nankasi, Alon Atuhaire, Candia Rowell, Annet Namukuta, Asmin Mohamed, Moses Adriko, Poppy Lamberton, Edridah Tukahebwa, Kathryn J. Allan, Christina Faust

**Affiliations:** 1 School of Biodiversity, One Health, and Veterinary Medicine, University of Glasgow, Glasgow, UK; 2 Vector Control Division, Ministry of Health, Kampala, Uganda; 3 Pathobiology and Population Sciences, Royal Veterinary College, Hawkshead, UK; 4 Department of Vector Biology, Liverpool School of Tropical Medicine, Liverpool, UK

**Keywords:** *Calodium hepaticum*, land cover change, mitogenome, zoonosis

## Abstract

*Calodium hepaticum* (syn. *Capillaria hepatica*) is a generalist nematode that infects liver parenchyma of mammals worldwide and can cause human infections. Prevalence ranges from 0% to 100% in wildlife across small geographic areas, making it an ideal parasite for understanding ecological drivers of variation given intensive land use or cover change. Here, we quantify prevalence of *Calodium hepaticum* and present initial surveys of synanthropic small mammals. Cross-sectional rodent trapping was conducted within and around households over 2 consecutive dry seasons in 4 villages with differing land cover. DNA was extracted from liver tissue and the 18s rRNA gene of *C. hepaticum* was amplified to confirm presence of *C. hepaticum.* Landscape structural diversity was classified by tree crown density and mean canopy height derived from 30 m LiDAR data within a 0.5 km buffer. Multivariable binomial generalized linear models were fit to *C. hepaticum* prevalence. *Calodium hepaticum* infection was common (overall 34.5%, CI 95%: 27.9–41.0) and found in rodent and shrew species inside and outside residences. We observe village-level differences in prevalence (18.2–75.0%), with higher *C. hepaticum* prevalence associated with lower relative proportion of native rodent species to *Rattus rattus* (adjusted OR = 0.55, CI 95%: 0.33–0.92). Host diversity appears to be protective against parasite prevalence. Differences in molecular and gross parasitological identification highlight challenges in diagnosis and a need for more specialized molecular tools. Further investigation is required to understand individual host and community variation in pathogen infection intensity and implications for zoonotic risk.

## Graphical abstract

**Figure:**
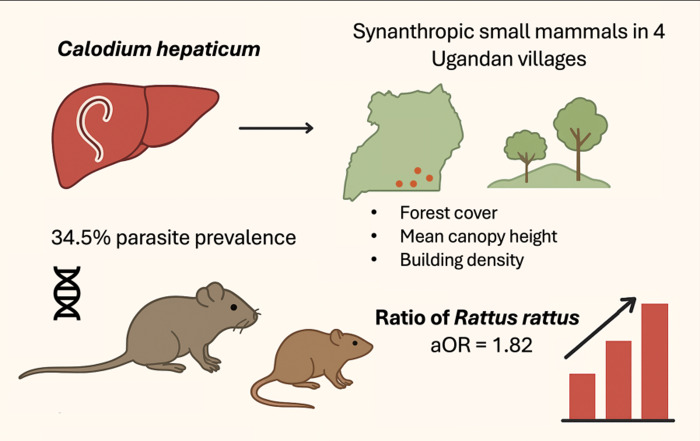

## Introduction

Small mammals, including rodents and shrews, are reservoirs of zoonotic pathogens and play critical roles in the dispersal and transmission of pathogens. Rodents and shrews host pathogens related to over 60 diseases of public health concern, variably caused by bacteria, helminths, protozoans and viruses (Han et al., 2015; Mangombi et al., 2021). Land use change, for example urbanization or agricultural intensification, disrupts social and ecological systems and is regarded as a critical driver of pathogen emergence (Jones et al., 2013; LaDeau et al., 2015). A key barrier to understanding emerging zoonotic disease is understanding the environmental conditions that link land cover change, small mammal ecology and pathogen dynamics.

Many rodent-associated ectoparasites and endoparasites have implications for veterinary and public health (Isaac et al., 2018). Zoonotic rodent-borne pathogens can be transmitted by ectoparasites, including fleas as the vector of plague (*Yersinia pestis*) (Adjemian et al., 2007) (Zimba et al., 2019) and tick-associated febrile diseases such as tick-borne relapsing fever (TBRF) and tularaemia (Dahmana et al., 2020). Rodent endoparasites can also contribute human or animal health problems, for example, contributing to the maintenance of *Toxoplasma gondii*, a parasite that causes disease in humans, cats, small ruminants and other mammals within an ecosystem (Dubey et al., 2021). Small mammals have been shown to harbour diverse parasites in localized sites, for example in Nigeria, where gross pathological investigation of endoparasites found small mammals to be infected with 12 helminth taxa (including *Strongyloides* sp., *Trichosomoides* sp. *and Trichuris* sp.) and 6 protozoan parasites (including *Toxoplasma gondii*) (Isaac et al., 2018; Boundenga et al., 2019). Rodents are an important reservoir for human zoonotic schistosomiasis in West Africa (Catalano et al., 2018) and are an important refuge of *Schistosoma japonicum* in Southeast Asia, reducing public health gains made through treatment programs (Zou et al., 2020). Cataloguing and quantifying the burden of pathogens in wild rodent hosts is therefore crucial for informing interventions.

In addition to harbouring important zoonoses, many small mammal species are highly adaptive, anthrophilic and thrive in human-altered landscapes. Global analysis has shown that the proportion of wildlife that host zoonotic pathogens increases with establishment of agriculture and land cover change, with particularly strong effect for rodent taxa (Gibb et al., 2020). Many rodent and shrew species have fast reproduction cycles and an early time to maturation, equating to rapid population turnover. Highly fluctuating populations and periods of high population density have been posited as a predictor of transmission risk and pathogen dispersal in rodent reservoirs, particularly for habitat generalist species (Ecke et al., 2022). In East Africa, invasive and native species of rodents and shrews occur in human-modified habitats in high abundances and host a diversity of zoonotic pathogens (e.g., (Halliday et al., 2013; Ogola et al., 2021; Rasoamalala et al., 2025)). Understanding if specific host assemblages and associated land uses facilitate higher pathogen transmission is important for predicting disease risk and informing control strategies.

A study was conducted to characterize a generalist nematode parasite *Calodium hepaticum* (syn. *Capillaria hepatica*) in small mammal communities in eastern Uganda*. Calodium hepaticum* has an extremely broad host range: recorded in over 90 Muroidean rodent species and around 70 species of mammals, including carnivores, shrews and primates (Fuehrer, 2013a,b). *Calodium hepaticum* causes hepatic calodiasis (syn. capillariasis) in heavily infected hosts. Adult worms develop in liver parenchyma and survive for approximately 6 weeks, with peak egg production at 40 days post infection (Oliveira and Andrade, 2001). Unembryonated eggs can be released into the environment through faecal samples of infected hosts (Chieffi et al., 1891), but the hypothesized main route of release is through death or consumption of the infected host (Spratt and Singleton, 2008). Eggs enter the environment through degradation of infected corpses or are mechanically passed through the gut of predator (Momma, 1930; Farhang-Azad, 1977; Conlogue et al., 1979; Wright, 2011). To become infectious, the egg must embryonate in the environment, which is dependent on temperature and humidity (Luttermoser, 1938; Spratt and Singleton, 2008; Wright, 2011). The embryonated eggs are then ingested by the rodent definitive host, or occasionally human hosts, then hatch and migrate to liver to continue the life cycle. The parasite is primarily diagnosed through gross observations of lesions on the liver and morphological identification of eggs, but more recently PCR primers have been developed to identify infections (Manor et al., 2021).

*Calodium hepaticum* has been found globally, but infection intensities and prevalence are highly variable. Typical of helminths, often there are a few heavily infected individuals with the majority having lighter infections (Conlogue et al., 1979; Reperant and Deplazes 2005, Sandy et al., 2024). Infection rates can be higher in mature or older hosts (Chieffi et al., 1891; Sinniah et al., 1979; Ceruti et al., 2001; Rothenburger et al., 2014) and has been associated with injuries, potentially associated with more aggressive individuals (Rothenburger et al., 2014). Prevalence has been positively correlated with small mammal host density in some studies (Davis, 1951; Childs et al., 1988; Meagher, 1999) and can range from 0% to 100% in populations on small spatial scales, even city blocks (Rothenburger et al., 2014). Studies have also linked *C. hepaticum* prevalence to habitat type (Davis, 1951; Childs et al., 1988; Roberts et al., 1992). The main hypothesis for this observed variation in prevalence across habitats is that microclimate conditions affecting embryonation of eggs and subsequent transmission.

Human infection with *C. hepaticum* has been recorded (Fuehrer et al., 2011), presenting as a hepatic calodiasis associated with poor sanitation and low socioeconomic indices. Although only reported sporadically, lack of distinct clinical signs and appropriate diagnostics suggests human cases may be underreported (Camargo et al., 2010; Gonçalves et al., 2012), human risk from wildlife populations requires investigation of a myriad of complex features tailored to local ecological context. These include infection prevalence in reservoirs and host species abundance, key determinants of the force of infection that are prerequisites for transmission of pathogens into human populations (Lloyd-Smith et al., 2009; Murray and Daszak, 2013). In vector-borne diseases, high prevalence of a pathogen in wildlife reservoirs has been linked to higher transmission risk to human populations (Johnson et al., 2023). For environmentally transmitted rodent-borne pathogens such as *Leptospira*, high household ‘rattiness’ – a composite proxy for rodent abundance – was found to be the factor most associated with higher infection risk for human residents (Eyre et al., 2022). However, further statistical modelling found that environmental variables such as vegetative land cover and household flooding were more influential than either rodent abundance or individual rodent pathogen shedding for human transmission risk (Soni et al., 2024). Other factors, such as infectious dose and human-wildlife contact patterns, will also be essential to understand transmission but fall outside the current scope.

A survey of *C. hepaticum* infection in small mammals was conducted in eastern Uganda across a heterogenous landscape of increasing human population and concomitant changes in land cover composition. The aim was to quantify infection prevalence and investigate the relationship with host factors (species, diversity, relative abundance) and landscape factors (forest cover, building density) to understand local drivers of infection.

## Materials and methods

### Site selection and household-level data

Infection of *C. hepaticum* was quantified in small mammals in sites in eastern Uganda, within 33° 17’ 0.49255” N, 0° 13’ 34.6728”W and 33° 58’ 40.7611056006” N, 0° 44’ 4.9775928003” E. Climate is mainly tropical, characterized by bimodal wet seasons typically occurring from March–May and September–November (Kizza et al., 2009).

**Figure 1.**
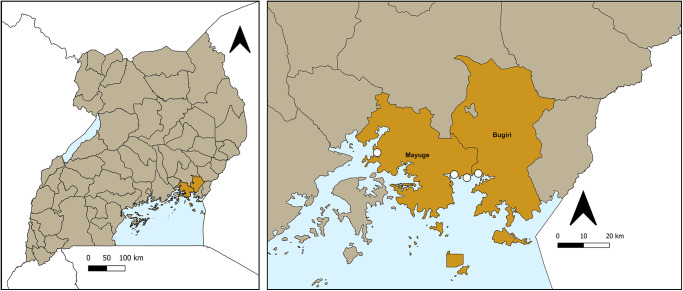
Map of study locations. (Left) Mayuge and Bugiri Districts in eastern Uganda. (Right) Study sites (white points) in Mayuge and Bugiri, adjacent to Lake Victoria. Figure 1 long description.

Live trapping was carried out in four villages across 2 dry seasons in July 2018 and February 2019 in Mayuge and Bugiri Districts, eastern Uganda (Figure 1). Villages ranged in density of households and forest cover and loss (Table S1) and were chosen to represent a spectrum of land uses: fishing boat landing sites (Bugoto), small holder agriculture (Musubi), and timber plantations (Walumbe, Waka Waka). All sites had agricultural land and were adjacent to Lake Victoria but differed in the primary land cover and use. Trapping and sampling protocols were reviewed and approved by the Vector Control Division Research Ethics Committee (VCDREC095), Ugandan National Council for Science and Technology (NS-622) and the University of Glasgow’s School of Veterinary Medicine Ethics (27a/18). A minimum of 10 households were recruited from each village. In 2018, questionnaires were administered to households to ascertain observed levels of rodent infestations (Table S2).

Within each household, a target of 3 traps was placed indoors and 2 traps were placed outdoors. A mixture of large Sherman traps (HB Sherman Traps, Tallahassee, USA. Dimensions: 7.6 × 8.9 x 22.9 cm; n = 80), medium Tomahawk (Tomahawk Live Trap, Hazelhurst, USA. Model 602 (dimensions 12.7 × 12.7 × 40.6 cm; *n* = 20) and local handmade treadle traps (here called Tomahawk-sized treadle traps; multiple captures; approximate dimensions 15 × 13 × 40 cm; *n* = 6) were used. Placement and number of traps was modified based on homeowner agreements. For each household, traps were left open and baited for 3–5 consecutive nights and checked first thing in the morning. Full traps were taken to a central processing unit for sample collection. Actual sampling effort varied between households, villages and sessions due to logistical constraints.

### Individual specimen collection

Full traps were taken to a central processing area for humane euthanasia and *post-mortems*. Individuals were euthanized via chloroform overdose and confirmation of death was by cervical dislocation. Morphometric measurements (head-body length, tail length, right ear and right hind foot) and weight were taken prior to dissection. Individuals were identified to species using morphology; however, some genera (*Mastomys, Mus, Crocidura)* are difficult to differentiate to species based on morphology alone, so molecular tools were used to determine species (see *Laboratory Analysis*) (Monadjem et al. 2015). All liver lobes were inspected for lesions and presence of *C. hepaticum*. Liver tissue was also stored in 70% ethanol with nuclease-free water for downstream laboratory analysis.

### Environmental classification

Satellite-derived remote sensing datasets were used to assemble local landscape features and human metrics (Table 1). Gridded UN-adjusted human population estimates were assembled at 1 km resolution (WorldPop, 2018). Counts of building footprints were extracted from Google Open Buildings at each site as a proxy for household density and urban environments (Sirko et al., 2021). Elevation data were obtained from NASA SRTM 90 m Digital Elevation Database v4.1 (CGIAR-CSI) (Jarvis et al., 2008), and broad climatic trends (min/max temperature, precipitation) were assembled from BioClim (Fick and Hijmans, 2017). Given extensive conversion of agricultural land, tree cover was derived from Hanson’s Global Forest Watch (30 m) (Hansen et al., 2013) contemporaneously and with a time lag of 2, 5 and 10 years prior to trapping. Classification of tree cover was examined at multiple thresholds, ranging from conservative (≥50% of crown density per pixel) to modest thresholds (≥30) (Figure S1). Mean canopy height (metres) was derived from Global Ecosystem Dynamics Investigation (Potapov et al., 2021a) using LiDAR measurements at 30 m resolution. Proportion of cropland in 2019 was extracted from a dataset of global cropland expansion derived from Landsat time-series data at 30 m resolution (Potapov et al., 2021b). To protect individual household identity, village centroids were taken as the mean coordinates of geolocated households and used to estimate spatial data. Environmental and anthropogenic covariates were extracted around village centroids within buffer radii of 0.5, 1, 2, 5 and 10 km and mean values are detailed in Table S1.

**Table 1.** Summary of environmental and anthropogenic covariates scales and source Table 1 long description.

Covariate	Temporal scale	Spatial scale	Source
Elevation (m)	2000	1 km	SRTM Version 4 (Jarvis et al., 2008)
Temperature (°C)	Month	1 km	WorldClim 2 (Fick and Hijmans, 2017)
Forest cover (count)	2009–2019	30 m	Global Maps of Forest Cover Change (Hansen et al., 2013)
Canopy height (m)	2019	30 m	Global Forest Canopy Height through GEDI (Potapov et al., 2021a)
Population density (p km^−2^)	2019	1 km	WorldPop UN-adjusted Population Density (WorldPop, 2018)
Building density (count)	2023	1 km	Google OpenBuildings (Sirko et al., 2021)
Cropland (%)	2019	30 m	Global Maps of Cropland Extent using LandSat (Potapov et al., 2021b)

### Laboratory analysis

DNA was extracted for host species confirmation and *C. hepaticum* molecular diagnosis. DNA was extracted from 25 mg of liver tissue with QIAgen Tissue and Blood Kit (Qiagen Ltd. Hilden, Germany) using manufacturer’s instructions with a 90-min incubation with proteinase K and eluted into a final volume of 200 μL. Extracted DNA was quantified using Qubit Broad Range Assay kits (Thermo Fisher Scientific, USA). For species, host cytochrome b (cytb) DNA was amplified using established protocols (Schlegel et al., 2012). Bands were visualized on 1.0% agarose gels and bands matching the ∼900 bp band were shipped to the UK and submitted for Sanger Sequencing with forward primers. A subset was also submitted with reverse primers. Resulting sequence traces were checked for clarity and trimmed with UGENE (Okonechnikov et al., 2012).

To identify presence/absence of *C. hepaticum* DNA, the 18s rRNA gene of *C. hepaticum* gene was amplified using previously published primers (Williams et al., 2020). PCR conditions were adapted and optimized for MgCl_2_, primer concentration and thermal profile to increase specificity and sensitivity. The final PCR thermal profile was an initial denaturation at 94 ^o^C for 5 min, then 35 cycles (denaturation at 94 ^o^C for 30 sec, annealing at 59 ^o^C for 30 sec, extension 72 ^o^C for 30 sec), then final extension at 72 ^o^C for 2 min. Final PCR was performed using the following master mix in 20 μL total reaction volumes: 10x PCR buffer, 1.5 mM MgCl_2_, 1 U Taq DNA polymerase (Invitrogen™ 18038026), 0.2 mM of each dNTP (NEB™ N0447S), 200 nM forward primer, 200 nM reverse primer, template DNA.

Initial PCR screening with liver DNA revealed a high incidence of very faint PCR bands (*n* = 29/203). Several of these samples had observable parasites during necropsy suggesting the initial PCR screen lacked sensitivity (Dataset 1). As liver tissue can have a high proportion of inhibitors that can affect amplification of DNA (Al-Soud et al., 2005), DNA was diluted 1:10 in nuclease-free water and PCRs were re-run. Molecular presence of *C. hepaticum* was conservatively estimated as a strong signal on either undiluted or 1:10 diluted DNA or a faint signal on both. Individuals with a faint signal on only one protocol were considered to be a false positive result and treated as a negative for subsequent analyses. A subset of PCR product from strong bands (*n* = 8) were sent for sequencing at DNA Sequencing and Services, MRC PPU, to confirm presence of C. *hepaticum.* The quality of these sequences was insufficient for deposition into GenBank.

No reference genome or mitogenome currently exists for *C. hepaticum.* To address this gap, a *C. hepaticum* worm dissected from a *R. rattus* in 2019 was prepared for shotgun sequencing. DNA extraction followed manufacturer’s instructions for Beckman Coulter 280 Agencourt DNAdvance gDNA purification kit and to increase yield, a single cell Illustra GenomiPhi DNA amplification kit was used (GE Healthcare Life Sciences, USA). DNA was submitted for sequencing through a commercial supplier and was sequenced on an Illumina NovaSeq 6000, aiming for 2 M paired-end reads. This depth was targeted to attempt to reassemble the mitochondrial genome.

### Data analysis

Adjusted trap success was used as a measure of relative abundance of small mammals for each village in each trapping session, calculated as total animals caught (*n*) as a proportion of trap nights corrected for misfired traps (number of traps set × number of nights – sprung traps * 0.5) (Nelson and Clark, 1973) and compared between village, household and trapping method. To assess whether sampling effort was sufficient to characterize host diversity, species accumulation curves were calculated for each site, for each trapping season, and overall. In addition, the proportion of households with small mammals was calculated as a general measure of household infestation. Household trap success was compared to qualitative surveys on household rodent control practices and lived experience of rodent contact (Table S2).

Prevalence of zoonotic hepatic nematode *C. hepaticum* were then calculated per village per year as the overall number of rodents or small mammals with molecular confirmation of *C. hepaticum* (according to above diagnostic criteria) out of number tested by molecular methods. Binomial confidence intervals for point prevalence estimates were calculated using the package DescTools (version 0.99.60) and used to compare the proportion of *C. hepaticum* infection between species, and between sex within-species. Statistical analysis was performed in R version 4.5.1 (2025-06-13) (R Core Team, 2021)

To assess village-level difference in prevalence, binomial confidence intervals at a 95% threshold were calculated around point estimates of village-level prevalence, as defined above. To assess possible environmental or human correlates of prevalence, bivariable analyses were conducted independently for each explanatory variable within a 0.5 km buffer. Coefficients from binomial generalized linear model (GLM) were fit using the package stats (version 4.5.1) in R and coefficients exponentiated to calculate odds ratios (OR). P values for the strength of evidence for independent variables were assessed using likelihood ratio tests (LRT). Variables were assessed for inclusion in the multivariable model based on a criterion of *p* > 0.2 for bivariable models (Table S3), conservatively set to ensure all potentially associated variables were included. Spearman’s rank tests were conducted on variables to observe correlation (Figure S2). To reduce high degrees of multicollinearity between independent variables, variance inflation factors (VIF) were examined following a stepwise procedure until only predictor variables meeting a moderate threshold of VIF ≤ 6 remained in the global model (Rogerson, 2001). Variables were assessed with a backwards selection strategy until a predetermined threshold of *α* < 0.05. Model fit was quantified using Nagelkerke’s *R*^2^ (DescTools, version 0.99.60) and residual diagnostics were calculated using the DHARMa package in R (version 0.4.7). Due to concerns about limited sample size and overdispersion, association between individual level infection probability (*n* = 203) and ecological covariates were assessed by fitting a binomial GLMM with a random effect for village using the R package lme4 (version 1.1-37) with model fit quantified using pseudo-R^2^ values (MuMIn, version 1.48.11).

### Bioinformatics

Raw reads were assembled using SPAdes v 3.15.5 (Prjibelski et al., 2020) default parameters. The most likely contig corresponding to the mitochondrial genome was identified by searching the assembled contigs with BLASTn (Altschul et al., 1990), using the mitochondrial genome of *Trichuris muris* [NC_028621.1] as a query. Similarity of the extracted putative mitochondrial genome to published sequence data was established through BLASTn searches of all records including ‘Nematoda (taxid:6231).’ The putative mitochondrial genome was used as input for the MITOS annotation webserver (Bernt et al., 2013). Finally, the resulting annotated COX1 (COI, Cytochrome c oxidase subunit I) gene was manually blasted against NCBI GenBank using *C. hepaticum* (taxid:1975702) to check similarity to published sequences.

## Results

### Small mammal trapping success and diversity at different sites

Four villages were included, selected from Mayuge District and Bugiri District in Eastern Uganda. Cross-sectional sampling (trapping over a single dry season) was conducted in 2 villages (Bugoto and Walumbe) in 2018, whilst in Musubi and Waka Waka repeat sampling was conducted over 2 consecutive dry seasons in 2018 and 2019. A summary of selected village-level trapping detail is given in Table 2. Traps were set in 98 participating households. 224 small mammals were trapped across the 4 villages over 2 dry seasons. Adjusted trap success by village and season ranged from 10.8% to 37.9% (Table 2). Small mammals were caught in 76.5% of households overall (N = 75/98), ranging from 53.8% of households in Bugoto to 80.8% of households in Waka Waka in 2019. Musubi saw an increase in proportion of households with small mammals from 55.6% in 2018 to 77.3% in 2019. A mean of 1.6 small mammals were trapped per household, ranging from 0 to a maximum of 6 in a single house.

**Table 2.** Trapping data and adjusted trap success by site and session Table 2 long description.

	Bugoto	Musubi		Walumbe	Waka Waka			
	2018	2018	2019	2018	2018	2019	Mean	SD
Sampling nights per village (*n*)	5	5	3	3	3	3	3.67	1.03
Total household trap nights per village (*n*)	27	84	66	66	67	72	63.67	19.23
Adjusted trap nights per village (*n*)	29.0	347.0	276.5	284.5	289.0	292.0	253.00	112.58
Total number full traps (*n*)	11	38	30	46	35	51	35.17	35.17
Adjusted trap success (%)	37.9%	11.0%	10.8%	16.2%	12.1%	17.5%	17.7%	10.4%
Total trapped (*n*)	18	44	30	46	35	51	37.33	12.16
Households with successful trapping (%)	53.8%	55.6%	77.3%	79.2%	75.0%	80.8%	70.3%	12.1%
Shannon Index (*H*)	0.0	1.16	1.55	2.15	1.01	1.30	1.20	0.71
Number of participating households	13	27	22	24	24	26	22.67	5.05

Household level surveys were conducted to assess perceptions of household infestation levels (*N* = 81). Nearly every household saw rodents in households, with many (71.6%, *N* = 58/71) reporting seeing rodents daily. Overall, 80.2% of households surveyed engaged in pest control (*N* = 65/81), and 75.3% of households used chemical methods (*N* = 61/81), consistent across villages (Table S2). Of households using chemical pest control, the most reported type was indometacin tablets, brand name Indocid (*N* = 44/61). Other chemical methods include Fuko-Kil (*N* = 3/61), and Rat-Rat (*N* = 2/61) and unidentified rodenticide pastes or powders (*N* = 15/61).

*Post-mortems* and pathogen screening was conducted on 234 small mammals in total, including small mammals that were brought to the study in non-standard traps and cases of multiple trap occupancy (*N* = 10) (Table 3, Dataset 2). Of these, 50.4% were female (N = 118/234) and 44.4% were male (*N* = 104/234), with 12 individuals not sexed. The majority (73.1%; *N* = 171/234) were classified as sexually mature based on external features. Of the 234 small mammals captured, 135 were caught in Sherman traps (57.7%), compared to 87 in Tomahawk or Tomahawk-sized treadle traps (37.2%).

**Table 3.** Summary of characteristics of rodents and shrews table (*N* = 234) Table 3 long description.

		Bugoto	Musubi		Walumbe	Waka Waka	
Category	**Total (%)**^a^	**2018**^a^	**2018**^a^	**2019**^a^	**2018**^a^	**2018**^a^	**2019**^a^
Sex							
Female	118 (50.4)	10 (55.6)	22 (47.8)	10 (32.3)	20 (43.5)	21 (58.3)	35 (61.4)
Male	104 (44.4)	8 (44.4)	23 (50.0)	18 (58.1)	21 (45.7)	13 (36.1)	21 (36.8)
**Genera**							
*Rattus* spp.	141 (60.2)	18 (100.0)	31 (67.4)	17 (54.8)	34 (73.9)	21 (58.3)	20 (35.1)
*Mus* spp.	31 (13.2)	0	1 (2.2)	0	2 (4.3)	8 (22.2)	20 (35.1)
*Mastomys* spp.	17 (7.2)	0	2 (4.3)	2 (6.5)	4 (8.7)	0	9 (15.8)
*Arvicanthis* spp.	10 (4.2)	0	3 (6.5)	3 (9.7)	0	0	4 (7.0)
*Lemniscomys* spp.	1 (0.4)	0	0	1 (3.2)	0	0	0
*Crocidura* spp.	34 (14.5)	0	9 (19.6)	8 (25.8)	6 (13.0)	7 (19.4)	4 (7.0)
**Trap type**							
Sherman	135 (57.7)	0	25 (10.7)	22 (9.4)	28 (12.0)	19 (8.1)	41 17.5)
Tomahawk	87 (37.2)	16 (6.8)	19 (8.1)	9 (3.8)	18 (7.7)	16 (6.8)	9 (3.8)
Treadle	12 (5.1)	2 (11.1)	2 (4.3)	0	0	1 (2.8)	7 (12.3)
**Location**							
Indoor	183 (78.2)	18 (100.0)	33 (71.7)	20 (64.5)	39 (84.8)	32 (88.9)	41 (71.9)
Outdoor	51 (21.8)	0	13 (28.3)	11 (35.5)	7 (15.2)	4 (11.1)	16 (28.1)
**Total (%)**^b^	234 (100.0)	18 (7.7)	44 + 2^c^ (19.7)	30 + 1 (13.2)	46 (19.7)	35 + 1 (15.4)	51 + 6 (24.4)

a Column percentage.

b Row percentage.

c Additional mammals brought in buckets and cases of multiple per trap.

**Figure 2.**
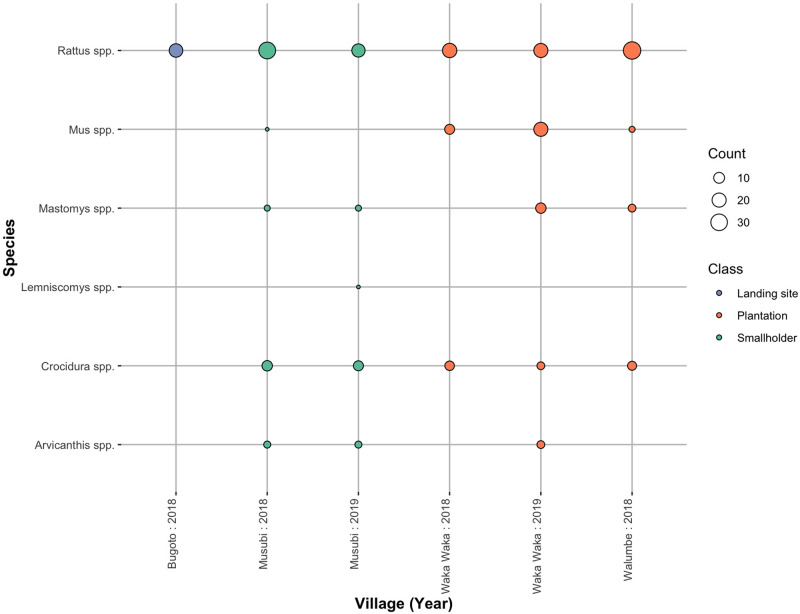
Bubble plot of small mammal genus and diversity across study site and year, coloured by land cover classification. (*N* = 234). Figure 2 long description.

Captured small mammals belonged to 6 distinct genera, primarily rodents (*Rattus* spp., *Mus* spp., *Mastomys* spp., *Arvicanthis* spp., *Lemniscomys* spp.) as well as shrews (*Crocidura* spp.) (Table 2). Distribution of genera according to study site and trapping session is illustrated in Figure 2 to include all individuals. Distribution by trap location and at species level is included in Supplementary Information (Figure S3). *Rattus rattus* was the most commonly trapped (*N* = 141/234, 60.3%), followed collectively by shrews (*Crocidura* spp.: *N* = 34/234, 14.4%) and house and pygmy mice (*Mus* spp.: *N* = 31/123, 13.2%). Less common were multimammate mice (*Mastomys* spp.: 17/234, 7.3%) and unstriped grass mice (*Arvicanthis niloticus: N* = 10/234, 4.3%), and only one typical striped grass mouse (*Lemniscomys striatus: N* = 1/234, 0.4%) was captured. Because morphological identification of species is not possible for all genera in this region (particularly *Mastomys, Mus* and *Crocidura*; Monadjem et al. 2015), mitochondrial genes were also amplified and sequenced to attempt species identification. There were incomplete or non-conclusive sequences for 14 individuals, the remainder were identified to species via morphological or molecular methods (Dataset 1).

Species accumulation curves were examined for overall sampling and disaggregated by village, with the caveat that some individuals were speciated and some were only identified to genus level. The plateau in the rarefication curve occurred at approximately 80 households and 15 species, indicating that increased intensity and stratification of sampling in each village and trapping season would be required to saturate species diversity (Figure S4).

*Village-level prevalence and distribution of* Calodium hepaticum

Liver infection with *C. hepaticum* was diagnosed both by gross parasitological examination and by PCR of both undiluted and a 1:10 dilution of liver DNA. Overall prevalence differed according to detection method (Table 4). Liver pathology was detected in 29% of small mammal postmortems (68/234, CI 95%: 23.2–34.9). PCR on undiluted DNA yielded an overall prevalence of 44.8% in a subset of small mammal samples that were tested using molecular methods (91/203, CI 95%: 38.0–51.7), compared to a lower overall prevalence of 26.1% (53/203, CI 95%: 20.0–32.2) using a 1:10 dilution. These prevalence estimates include faint bands, which are likely false positives. True positive was considered to be a strong band on either PCR or a faint band on both PCRs. Overall prevalence was 34.5% (70/203, CI 95%: 27.9–41.0) (Table 4).

**Table 4.** Characteristics of rodents tested and number/percentage of confirmed *C. Hepaticum* infections. LP, liver pathology; X1, PCR positive, X10; PCR positive dilution; P, combined overall positive (1 strong or both faint) Table 4 long description.

		*N*	LP + (%)^a^	N^M^^b^	X1 + (%)^a^	X10 *+* (%)^a^	P + (%)^a^	CI 95%^c^
**Genus**	*Arvicanthis* spp.	10	6 (60.0)	9	3 (33.3)	5 (55.6)	4 (44.4)	(12.0–76.9)
	*Crocidura* spp.	31	1 (2.9)	29	12 (41.4)	10 (34.5)	8 (27.6)	(11.3–43.9)
	*Lemniscomys* spp.	1	0 (0.0)	1	0 (0.0)	0 (0.0)	0 (0.0)	–
	*Mastomys* spp.	17	7 (41.2)	15	7 (46.7)	6 (40.0)	9 (60.0)	(35.2–84.8)
	*Mus* spp.	20	4 (13.3)	29	9 (31.0)	2 (6.9)	7 (24.1)	(8.6–39.7)
	*Rattus* spp.	142	50 (35.2)	120	60 (50.0)	30 (25.0)	42 (35.0)	(26.5–43.5)
**Trap**	Sherman	135	21 (15.5)	124	50 (40.3)	28 (22.6)	38 (30.6)	(22.5–38.8)
	Tomahawk ^d^	87	45 (51.7)	68	38 (55.9)	21 (30.9)	30 (44.1)	(32.3–55.9)
	Misc	12	2 (16.7)	11	3 (27.3)	4 (36.4)	2 (18.2)	(0.0–41.0)
**Location**	Indoor	183	56 (30.6)	159	72 (45.3)	38 (28.9)	50 (31.4)	(23.3–38.7)
	Outdoor	51	12 (23.5)	44	19 (43.2)	15 (34.1)	20 (45.5)	(30.7–60.2)
**Site type**	Landing site	18	3 (16.6)	8	6 (75.0)	4 (50.0)	6 (75.0)	(45.0–100)
	Plantation	139	46 (33.1)	127	51 (40.2)	22 (17.3)	34 (26.8)	(19.1–34.5)
	Smallholder	77	19 (24.7)	68	34 (50.0)	27 (39.7)	30 (44.1)	(32.3–55.9)
**Village, Year**	Bugoto 2018	18	3 (16.7)	8	6 (75.0)	4 (50.0)	6 (75.0)	(45.0–100)
	Musubi 2018	46	15 (32.6)	37	23 (62.2)	21 (56.8)	23 (62.2)	(46.5–77.8)
	Musubi 2019	31	4 (12.9)	31	11 (35.5)	6 (19.4)	7 (22.6)	(7.9 – 37.3)
	Walumbe 2018	46	16 (34.8)	41	19 (46.3)	10 (24.4)	11 (26.8)	(13.3–40.4)
	Waka Waka 2018	36	13 (36.1)	31	19 (61.3)	6 (19.4)	13 (41.9)	(24.6–56.3)
	Waka Waka 2019	57	17 (29.8)	55	13 (23.6)	6 (10.9)	10 (18.2)	(8.0–28.4)
**Overall**		234	68 (29.1)	203	91 (44.8)	53 (26.1)	70 (34.5)	(27.9–41.0)

a Proportion of N positive for *C. hepaticum* (row %).

b Denominator (N^M^) small mammals tested by molecular methods.

c 95% confidence interval (CI 95%) calculated in R using count and sample size (binomial distribution).

d Tomahawk combined with Tomahawk-sized treadle traps.

**Figure 3.**
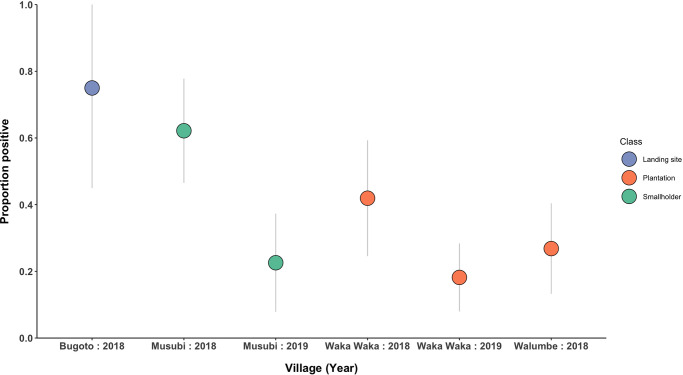
Proportion of small mammals infected by *C. hepaticum* by village site and trapping season. Infection status was determined by molecular detection in 1 or 2 assays. Colour coded by site landscape classification. Bars indicate 95% confidence intervals. (*N* = 203). Figure 3 long description.

Highest prevalence (overall combined positives) of *C. hepaticum* in small mammals was observed in Bugoto, 2018, with 75% of small mammals tested by molecular methods found to be infected (6/8, CI 95%: 45.0–100.0). Lower prevalence was observed in Waka Waka in 2019, with 10/55 small mammals testing positive (18.2%, CI 95%: 8.0–28.4) and an intermediate proportion of small mammals infected in Walumbe, 2018 (26.8%, 11/41, CI 95%: 13.3–40.4). Prevalence was notably higher in sites categorized as landing sites at 75% (6/18, CI 95%: 45.0–100.0) compared to forest plantations areas (26.8%, 34/139, CI 95%: 19.9–34.5). Infection prevalence in small mammals in Musubi fell between consecutive trapping seasons, with high infection prevalence of 62.2% in 2018 (23/37, CI 95%: 46.5–77.8) and only 22.6% in the following trapping season (23/37, CI 95%: 7.9–37.3). In Waka Waka, a similar temporal trend was observed, though not found to be significant at a 95% confidence threshold (Figure 3). Prevalence of *C. hepaticum* in small mammals does not appear to differ significantly between trapping location (inside/outside household), trap type or small mammal genus (Figure S5). Differences between villages and trapping seasons is not reflected in categorical description of site, suggesting other landscape or ecological features is driving differences in infection burden.

### Landscape effects on village-level infection prevalence

Landscape effects were first evaluated with bivariable generalized linear models to assess the association between individual village covariates and prevalence. A negative association between village level *C. hepaticum* infection prevalence and mean monthly climatic factors (maximum and minimum temperature), as well as weak associations with human population density estimates and absolute forest cover was found. A strong negative association was observed between the ratio of other genera to *R. rattus*, a proxy for small mammal community diversity between villages and seasons, and *C. hepaticum* prevalence. Following model selection, the best multivariable generalized linear model included the ratio of other small mammal genera to *R. rattus*, forest cover, and proportion of houses with rodents (Table 5). A significant strong negative association was retained between the ratio of all other small mammal genera to *R. rattus* and prevalence of *C. hepaticum.* This indicates that higher ratio of native species to *R. rattus* is associated with substantially lower odds of village-level *C. hepaticum* prevalence (aOR = 0. 55, CI 95%: 0.33–0.92, *p =* 0.02), or taking the inverse, that higher relative abundance *of R. rattus* is associated with substantially higher odds of *C. hepaticum* infection (aOR = 1.82, CI 95%: 1.23–3.03). The fitted model had a Nagelkerke *R*^2^ of 0.88, indicating a moderate-high proportion of variation in infection prevalence explained by the covariates and residual diagnostics did not show major deviations from uniformity nor evidence overdispersion (dispersion ratio = 1.26, *p* = 0.43). Additional individual-level GLMM further demonstrates a negative association between village-level ratio of other genera to *R. rattus* and individual-level probability of *C. hepaticum* and supports village level clustering consistent with spatial aggregation of infection (Table S4).

**Table 5.** Multivariable generalized linear model results for village-level prevalence of *C. Hepaticum* Table 5 long description.

	aOR	CI 95%		*P*-value^a^
Ratio (Other: *Rattus rattus*)	0.55	0.33	0.92	0.02109^b^
Forest cover (count)^b^	0.95	0.47	1.98	0.8843
Proportion of houses with rodents (%)	0.91	0.53	1.56	0.726

Signif. codes: 0 ‘***’ 0.001 ‘**’ 0.01 ‘*’ 0.05 ‘.’ 0.1 ‘.’ 1.

a Derived from likelihood ratio test (LRT).

b Threshold for forest = 30% tree canopy cover.

### Mitogenome recovery

Sequencing of an adult worm recovered an assembly of mitochondrial DNA. BLASTn query of the *Trichuris muris* mitochondrial genome against the final assembly reported a single hit (NC_028621.1), of 4159bp in length. A BLASTn search of this hit against Genbank: Nucleotide records for Nematoda returned 58 hits, 15 of which are reported in Table S5. The top 4 hits had query coverage of 89%, 97%, 63% and 50%, e-values of 0, and corresponded to *Pseudocapillaria* (MZ708958), *Aonchotheca* (NC_071371)*, Capillaria* (MH665363), and *Eucoleus* (NC_056391), respectively, all parasitic nematodes in the *Capillaridae* family. MITOS annotation identified 36 gene, tRNA and rRNA features (Dataset 3). The putative mitochondrial genome annotations were compared with those of *Trichuris muris* (NC_028621) and *Pseudocapillaria tomentosa* (MZ708958). The putative mitochondrial *C. hepaticum* was identical in feature order to that of *P. tomentosa.* Missing features with respect to *T. muris* and *P. tomentosa* were the *Atp8* gene and tRNA-Tyr, as well as an interrupted 16sRNA annotation.

The COI gene from the new mitochondrial genome was compared to all *C. hepaticum* records in NCBI Genbank (*n* = 3, KC355434, KC355435, MF962896). The top hit was MF962896 (91.27% sequence identity) and was a direct submission from a *R. rattus* in Argentina. Two records (KC355434, KC355435) were from the same *Arvicola terrestris* (water vole) from Switzerland and had lower sequence similarity (79.69%, 79.25%) (Guardone et al., 2013).

## Discussion

Land use change is known to disrupt ecological and social systems in ways that can affect pathogen dynamics. Parasite infection prevalence and intensity can be influenced by host community composition, host density, variation in susceptibility between host species or environmental features. A heavy parasite burden of *C. hepaticum* was observed, with high prevalence across diverse species, both inside and outside residences, and evidence of village-level spatial heterogeneity in prevalence. Strong links were identified between higher ratio of *R. rattus* to other native small mammal species to higher village-level prevalence, as well as observed lower prevalence in small-holder agriculture. Preliminary results offer evidence of a dilution effect, with low-moderate canopy cover sufficient to support higher overall species diversity adjacent to small-holder agriculture and timber plantations. This is the first study to screen synanthropic small mammals for *C. hepaticum* using molecular methods in East Africa. These findings provide insight into ecological mechanisms of maintenance and transmission for a zoonotic pathogen in the context of changing land use and land cover and indicate the need for further molecular and ecological studies characterizing *C. hepaticum* and associated pathogens across a landscape gradient.

Infection prevalence and host abundance are key prerequisites for spillover into human populations. Despite consistently high coverage of chemical pest control, high levels of household infestation and incursion were recorded in all villages. Higher levels of household incursion were recorded in 2019 compared to 2018, but only 2 sites were sampled in 2019. The temporal trend was consistent with loss in forest cover and the observation that proportion of forest cover is negatively correlated to household infestation (higher household infestation in areas with less forest cover). Although below thresholds for statistical significance, results are also indicative of an association between high household infestation (a metric for small mammal abundance) and higher prevalence, suggesting in this context that abundance may modulate prevalence in determining overall force of infection and have consequences for human risk. In support of this, a study found a heavily infected *Mastomys natalensis* in a home of a patient with hepatic calodiasis (Cochrane et al., 1957). While human risk for infection is expected to be tightly linked with infestation and prevalence, more studies are needed to demonstrate if household infestation with infected small mammals is sufficient, or if quantifying transmission at larger spatial scales is required to inform human risk.

In 4 sites across 2 dry seasons, 6 genera were captured: 5 rodent (*Mastomys, Rattus, Mus, Arvicanthis, Lemniscomys)* and 1 shrew (*Crocidura*). All sites recorded *Arvicanthis* spp., *Crocidura* spp., *Mastomys* spp., *Mus* spp. and *Rattus* spp., except for Bugoto, where exclusively *R. rattus* was identified. The focus on households, and land immediately surrounding households, may have limited the species that were detected. In the region there is a higher diversity of rodents in agricultural fields, including *Lophuromys* and *Dasymys* (Mayamba et al., 2019), that were not found due to sampling design. Adjusted trap success (ATS) showed a strong positive correlation to building density (ρ = 0.83), indicating higher trap success in areas with dense infrastructure, and a strong negative correlation coefficient with Shannon Index (H) (ρ = − 0.88) indicating higher trap success in sites dominated by fewer species. Bugoto is characterized as a ‘landing site,’ which denotes a village related to fishing trade, often urbanized, and densely populated. Bugoto exceeds the upper standard deviation for building density, and recent longitudinal studies suggest that a dense urban patch is likely to favour increased dominance of highly adapted species like *Rattus* spp. and local extirpation of other species via interspecific competition (Teitelbaum, 2024). It was also noted by inhabitants that when ‘stinky rats’ (a colloquialism for shrews, *Crocidura* spp.) are present, there is a noticeable absence of other rodent species. Whilst not a pattern observed here, this is supported by recent diet analysis and DNA metabarcoding of *Crocidura olivieri* that confirmed predation on *Mus musculus* (Galan et al., 2023).

Consistent with *C. hepaticum* being a generalist nematode parasite, *C. hepaticum* was observed in almost all species examined. Moderate infection prevalence was observed in native species: including *Mastomys* spp. (60.0%, CI 95%: 35.2–84.8) and African Giant White-toothed shrews (*Crocidura olivieri*, 27.6%, CI 95%: 11.3–43.9). Both species were found inside and outside households, suggesting they pose a risk to humans within households but can also link to small mammal assemblages in neighbouring fields and habitats. *Calodium hepaticum* has previously been identified in shrews in Europe, USA and Asia (Li et al., 2010; Fuehrer, 2013a; Miterpáková et al., 2024), but this is the first evidence of infection in African shrews. The only host without *C. hepaticum* was *Lemniscomys striatus*, but only one individual was trapped during the survey. Overall, ubiquitous infection of *C. hepaticum* was identified across all host genera and locations. The nematode parasite is common in this region in rodents and shrews, many of which are trapped within households, posing a risk for humans in these communities.

To identify infection with *C. hepaticum*, gross parasitological examination was first employed in the field and followed up with molecular diagnostics. Molecular detection, as expected, was higher than gross parasitological evaluation, but came with additional challenges. While the majority of individuals were screened molecularly, 13.2% of samples were omitted due to DNA degradation or sample loss during processing. There were difficulties using published primers to consistently amplify *Calodium* DNA, which another study from Hong Kong (Manor et al., 2021) also found. In order to address this, a mitogenome was generated from a worm isolated in the study region using shot-gun sequencing and de novo assembly. The mitogenome is highly divergent from published mitochondrial genomes and can be used in the future to design better primers. However, as observed in the COI region, the taxa shows substantial genetic divergence and baseline diversity is poorly understood. In recent years, additional genetic sequences have been made available (Buńkowska-Gawlik et al., 2017; Tamaru et al., 2025), which will improve the development of sensitive and specific diagnostics for this understudied nematode.

Clear differences in village-level *C. hepaticum* prevalence were observed between different sites. Land use and land cover heterogeneity is known to be linked to parasite infection prevalence in wildlife (Roberts et al., 1992; Gillespie and Chapman, 2008). It was hypothesized that expansion of agriculture might disrupt ecological equilibrium of rodent assemblages and result in higher pathogen burden. Instead, there was no detectable statistical relationship between village-level prevalence in rodents and recent forest loss, although it is important to note that the study was not designed to explicitly test for this relationship. Recent global meta-analyses find that habitat loss may not be a significant global driver of disease prevalence (Mahon et al., 2024) and only weak effects of forest loss on parasite prevalence (Heckley and Becker, 2025). Consensus dictates that parasite prevalence response to forest loss will be complex, context dependent, and contingent on parasite taxa and transmission mode (Gottdenker et al., 2014). However, by site description, prevalence was found to be significantly lower in sites adjacent to forest plantations (Waka Waka, Walumbe) compared to landing sites (Bugoto). Timber plantations may be affecting small mammal populations by supporting predators and act as ecological buffer against higher infection prevalence. While tree cover and canopy height provide an approximation of land cover composition meaningful for small mammal ecology, future work would be enriched by consideration of land cover configuration (connectivity, fragmentation, patch statistics) that influence rodent community diversity and abundance (Morales-Díaz et al., 2019) and pathogen dynamics (Teitelbaum, 2024).

Biodiversity and species community composition can have differential effects pathogen prevalence in a host population. In multivariable analyses, the only significant predictor of *C. hepaticum* prevalence to explain village-level differences was ratio of native small mammal species to *R. rattus*: with higher ratio of native species to *R. rattus* (more diverse species assemblage), *C. hepaticum* prevalence is lower. Theoretical frameworks show that in some contexts, higher species diversity (higher Shannon Index, H) is associated with higher intensity transmission but decreases in overall proportion of infectious individuals (lower overall prevalence) (Roche et al., 2012). Empirical longitudinal data on land consolidation and rodent-pathogen dynamics provides a link between rodent ecology, disease ecology and land use, with evidence that in addition to favouring a single dominant species (lower H), land consolidation results in higher pathogen prevalence in the newly dominant host species (Pei et al., 2024; Teitelbaum, 2024). Likewise, in Sierra Leone and Guinea, presence of *R. rattus* decreased prevalence of Lassa virus by competing with the dominant reservoir *M. natalensis* (Eskew et al., 2024). In this system, dominance of generalist species *R. rattus* may potentiate *C. hepaticum* burden through interspecies competition, while native species – supported by more heterogenous vegetation – creates a dilution effect (Keesing et al., 2006). However, this only represents 4 villages with 2 time-points, limiting the statistical power to infer meaningful relationships between environmental covariates and village-level differences in rodent abundance or parasite prevalence. In future analyses, more robust estimates of population prevalence could have been obtained with hidden Markov models to account for a latent observation process. Individual variation in pathogen load was not captured by the scope of the study. Understanding how local land use changes species composition and competition will be key to predicting pathogen burden.

## Conclusion

Identifying key drivers of pathogen infection prevalence and intensity in wildlife is essential to understand disease dispersal and transmission. Using both microscopic and molecular diagnostics, a broad host range of *C. hepaticum* was confirmed, identified in all sites and in all invasive and native species of rodents and shrews. Higher relative abundance of *R. rattus* appears to potentiate *C. hepaticum*, a concern for further exacerbation of zoonotic risk in more vulnerable landing site communities. Future work should focus on understanding the key ecological drivers of transmission for *C. hepaticum* and specific hazard to public health.

## Supporting information

10.1017/S0031182026101723.sm001 Johnson et al. supplementary material Johnson et al. supplementary material

## Data Availability

Data on household trap success (Dataset 1), details of *post-mortems* (Dataset 2), and mitochondria annotations (Dataset 3) are uploaded as supplementary tables. Sequence data from host (Accession numbers TBD) and parasite (PX206072) is deposited on GenBank.

## Supplementary material

The supplementary material for this article can be found at https://doi.org/10.1017/S0031182026101723.

## Table 1 Long description

The table provides a summary of various environmental and anthropogenic covariates, detailing their temporal and spatial scales along with their sources. Elevation data from 2000 is available at a 1 km spatial scale, sourced from SRTM Version 4. Temperature is measured monthly at a 1 km scale, sourced from WorldClim 2. Forest cover data spans from 2009 to 2019 at a 30 m scale, sourced from Global Maps of Forest Cover Change. Canopy height and cropland data from 2019 are available at a 30 m scale, sourced from GEDI and LandSat respectively. Population density and building density data from 2019 and 2023 are provided at a 1 km scale, sourced from WorldPop and Google OpenBuildings. The table highlights the diversity in temporal and spatial scales used in environmental data collection, reflecting the varying nature of each covariate's measurement and source.

Navigate back to Table 1.

## Table 2 Long description

The table presents trapping data and adjusted trap success across four sites, Bugoto, Musubi, Walumbe, and Waka Waka, for the years 2018 and 2019. Key metrics include sampling nights, total and adjusted trap nights, full traps, trap success rates, total trapped, household success rates, Shannon Index, and participating households. Bugoto in 2018 achieved the highest adjusted trap success at 37.9%, while Musubi in 2018 had the lowest at 11.0%. The number of sampling nights varied, with Bugoto and Musubi having the most at five nights each. The Shannon Index, indicating biodiversity, was highest in Waka Waka in 2018 at 2.15. Household participation was highest in Musubi in 2018 with 27 households. The data suggests variability in trapping success and biodiversity across different sites and years, with potential implications for ecological studies.

Navigate back to Table 2.

## Table 3 Long description

The table summarizes characteristics of 234 rodents and shrews, focusing on sex, genera, trap type, and location across different sites and years. Rattus spp. is the most prevalent genus, comprising 60.2% of the total, with a significant presence in all locations, especially indoors. Sherman traps are the most effective, capturing 57.7% of the total. Females slightly outnumber males, with 50.4% of the total. The data shows a higher capture rate indoors (78.2%) compared to outdoors. Mus spp. and Crocidura spp. are less common, with notable variations in capture rates across different sites and years. The table highlights the diversity and distribution of species, influenced by location and trap type.

Navigate back to Table 3.

## Table 4 Long description

The table measures the characteristics of rodents tested for C. hepaticum infections, including genus, trap type, location, site type, and village-year. Rattus spp. had the highest infection rate with 50% PCR positive, while Lemniscomys spp. showed no infections. Tomahawk traps were more effective than Sherman traps, with higher positive rates. Indoor locations had a higher number of infections compared to outdoor. Among site types, smallholder sites had a notable infection rate. The data suggests variability in infection rates based on genus, trap type, and location, with confidence intervals indicating the reliability of these findings.

Navigate back to Table 4.

## Table 5 Long description

The table presents results from a multivariable generalized linear model analyzing factors affecting village-level prevalence of C. Hepaticum. The most notable finding is the significant association between the ratio of other species to Rattus rattus, with an adjusted odds ratio of 0.55 and a p-value of 0.02109, indicating a potential inverse relationship. Forest cover and the proportion of houses with rodents show no significant association, with p-values of 0.8843 and 0.726 respectively. The confidence intervals for all variables suggest variability in the data, and the significance codes indicate the strength of the associations. The threshold for forest cover is defined as 30% tree canopy cover, which may influence the interpretation of forest-related data.

Navigate back to Table 5.

## Figure 1 Long description

The image consists of two maps. The left map shows Uganda with highlighted regions in the eastern part, specifically Mayuge and Bugiri Districts. The right map provides a detailed view of these districts, with study sites marked as white points. Both maps include scale bars indicating distances in kilometers and directional arrows pointing north. The districts are adjacent to Lake Victoria and the maps illustrate the geographical context of the study locations.

Navigate back to Figure 1.

## Figure 2 Long description

A bubble plot showing small mammal species distribution across different villages and years. The x-axis is labeled 'Village (Year)' with entries: Bugoye 2018, Mabira 2018, Mabira 2019, Mabira 2019, Wabia Wasa 2018, Wabia Wasa 2019 and Wabia Wasa 2019. The y-axis is labeled 'Species' with entries: Rattus spp., Mus spp., Mastomys spp., Lemniscomys spp., Crocidura spp. and Arvicanthis spp. Bubbles vary in size representing count, with sizes corresponding to 10, 20 and 30. The bubbles are colored by class: Landing site, Plantation and Smallholder.

Navigate back to Figure 2.

## Figure 3 Long description

The graph shows the proportion positive of small mammals infected by village and year. The x-axis is labeled 'Village (Year)' and includes Bugoto 2018, Musubi 2018, Musubi 2019, Waka Waka 2018, Waka Waka 2019 and Walumbe 2018. The y-axis is labeled 'Proportion positive' with values ranging from 0 to 1. Data points are categorized by class: landing site, plantation and smallholder. Bugoto 2018 shows the highest proportion positive, represented by a landing site category. Musubi 2018 and 2019 show lower proportions, categorized as smallholder. Waka Waka 2018 and 2019, categorized as plantation, show decreasing proportions. Walumbe 2018, also categorized as plantation, shows a lower proportion positive. Vertical lines indicate confidence intervals for each data point.

Navigate back to Figure 3.
