# Supplementary material for: Prevalence of zoonotic nematode Calodium hepaticum varies with small mammal community diversity across a heterogenous landscape in Eastern Uganda
Source: Parasitology. 2026 Feb 18;153(4):572–84. doi: 10.1017/S0031182026101723 (PMC13244227; doi:10.1017/S0031182026101723)
Supplement: Johnson et al. supplementary material [file S0031182026101723sup001.zip › SI_CAL_clean_20260113_for_submission.docx]

**Supplementary Information**

**Prevalence of zoonotic hepatic nematode varies with small mammal community diversity across a heterogenous landscape in Eastern Uganda**

Emilia Johnson^1^, Diana Ajambo^2^, Maria Capstick^1^, Moses Arinaitwe^2^, Olivia Ericsson^1^, Fred Besigye^2^, Jayna Raghwani^3^, Tristan P W Dennis^4^, Ronald Bogere^2^, Andrina Nankasi Barungi^2^, Alon Atuhire^2^, Candia Rowell^2^, Namukuta Annet^2^, Asmin Mohamed ^2^, Moses Adriko^2^, Poppy H L Lamberton^1^, Edridah Tukahebwa^2^, Kathryn J Allan^1^ and Christina L Faust^1^

^1^ University of Glasgow, School of Biodiversity, One Health, and Veterinary Medicine, Glasgow, UK

^2^ Ministry of Health, Vector Control Division, Kampala, Uganda

^3^ Royal Veterinary College, Pathobiology and Population Sciences, Hawkshead, UK

^4^ Liverpool School of Tropical Medicine, Department of Vector Biology, Liverpool, UK


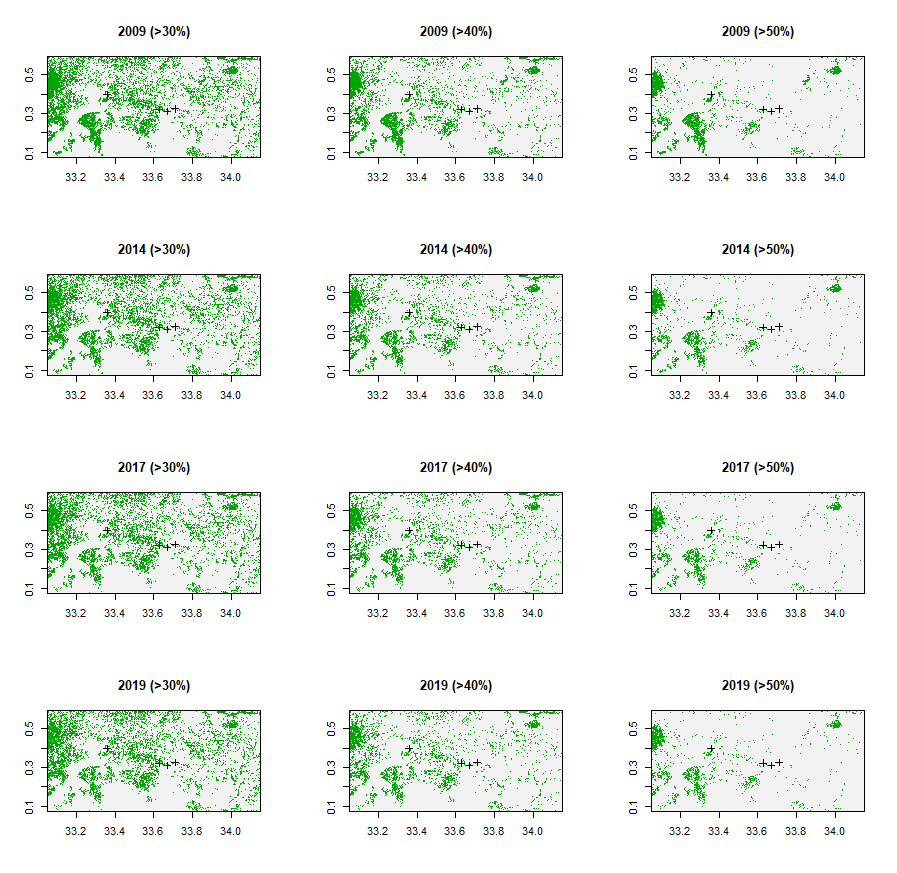


**Figure S1**: Forest (green) and non-forest (white) progressively lost in years 2009, 2014, 2017 and 2019 (plots top to bottom). Plots left to right show increasingly conservative definition of forest, according to proportion of canopy cover (>30%, >40% and >50% most conservatively).

**
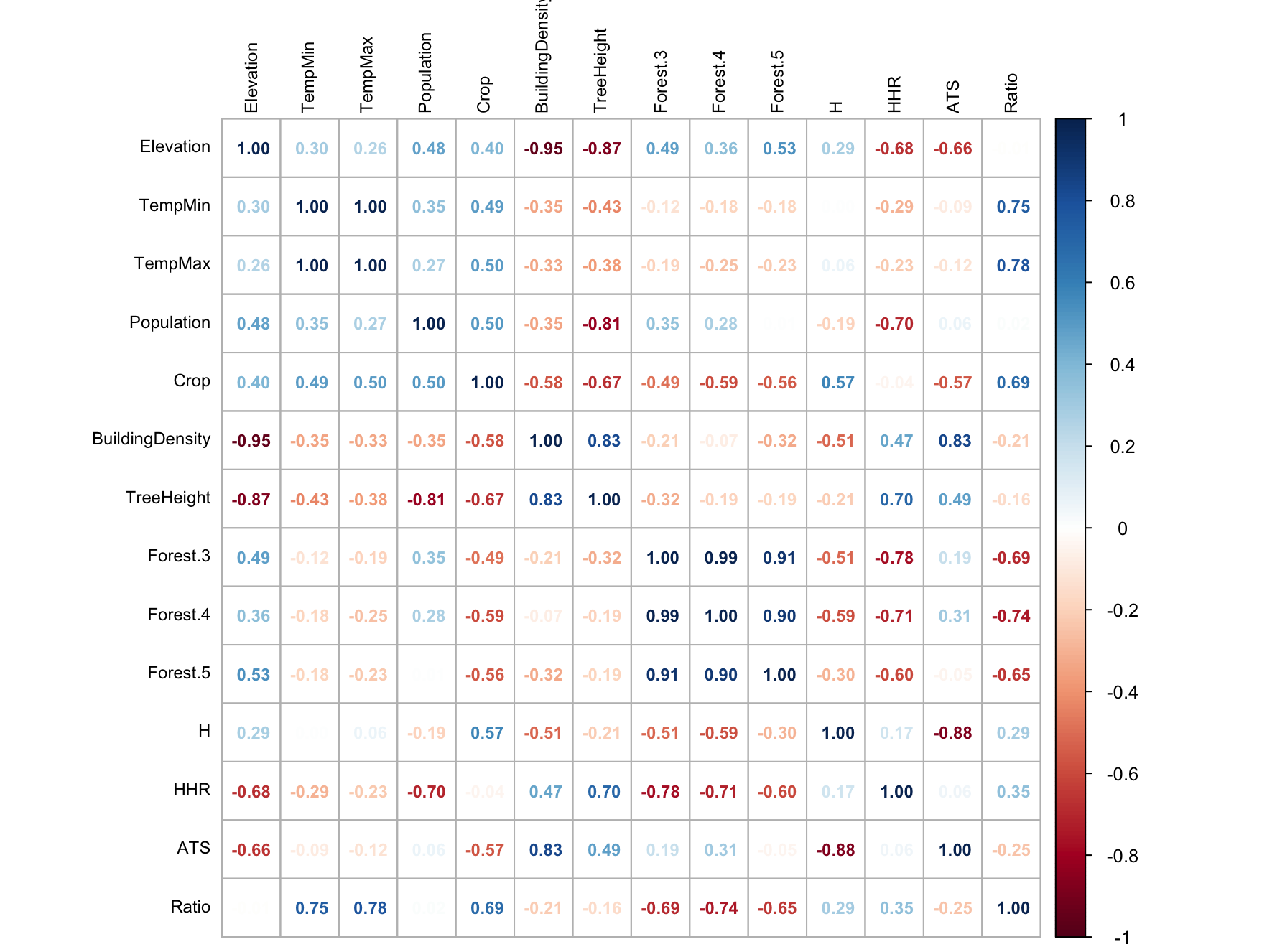
**

**Figure S2:** Spearman’s rank correlation matrix of environmental and ecological variables remaining after bivariable selection. H = Shannon Index for diversity; HHR = household rattiness (%); ATS = adjusted trap success.


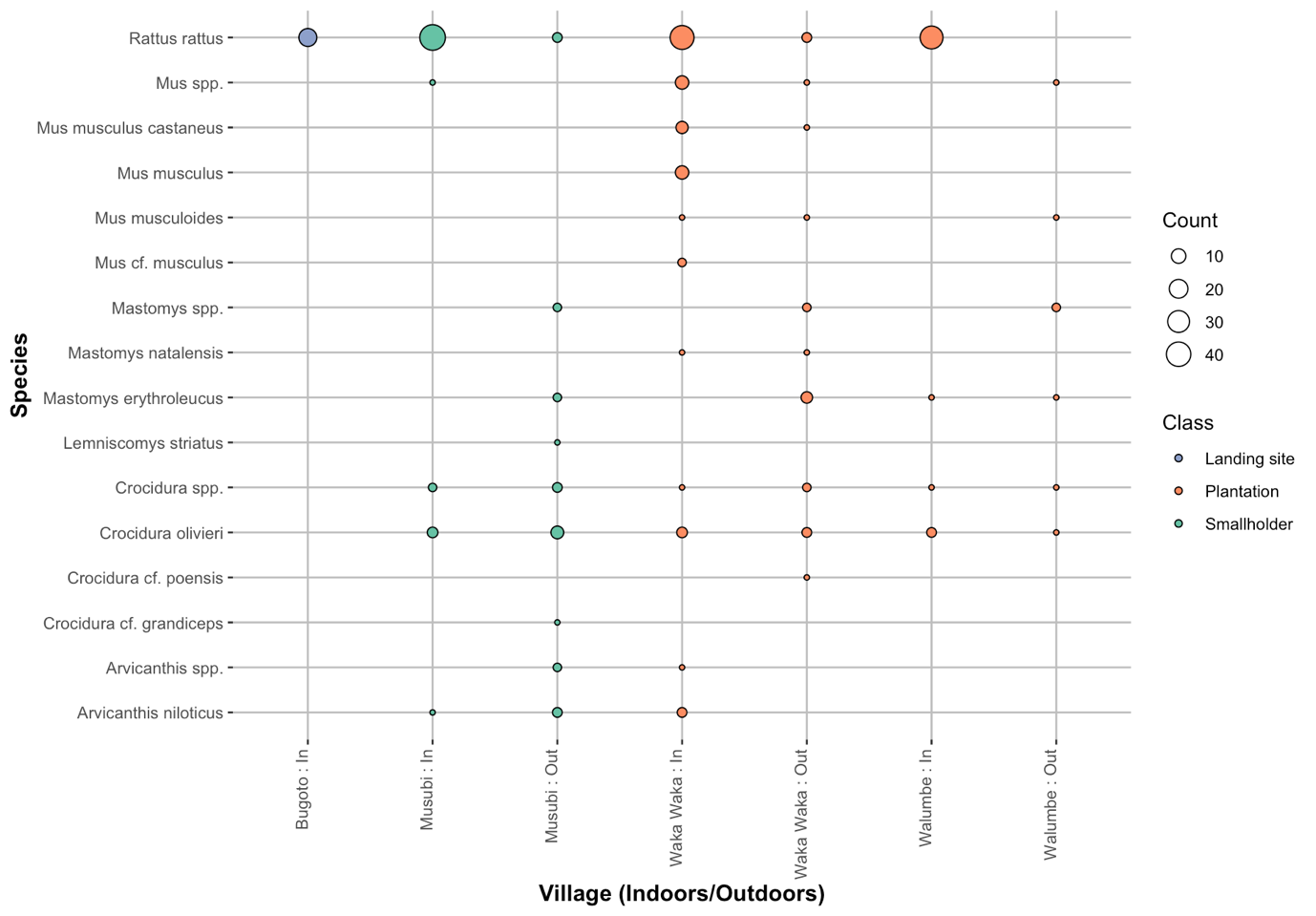


**Figure S3:** Bubble plot of species abundance by minimum classification of species/genera, village and whether small mammal was trapped inside or outside the household. Colour coded by land class.


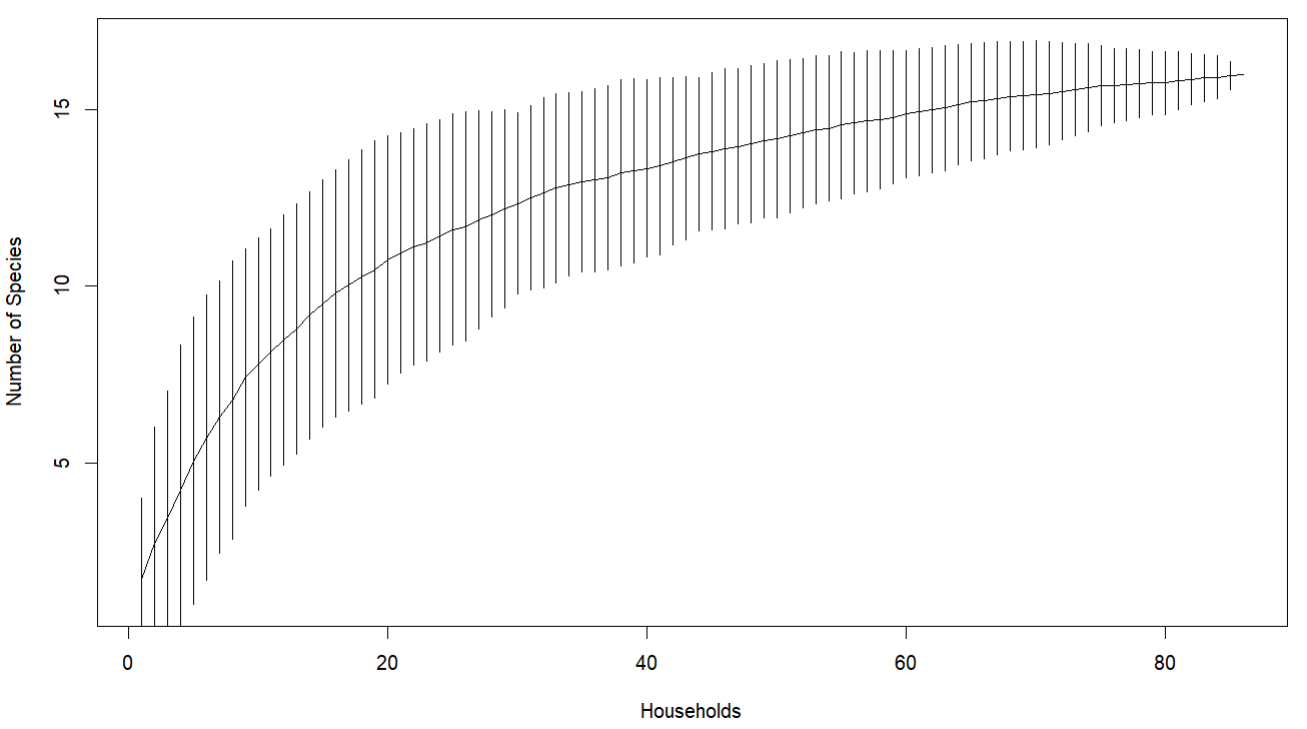


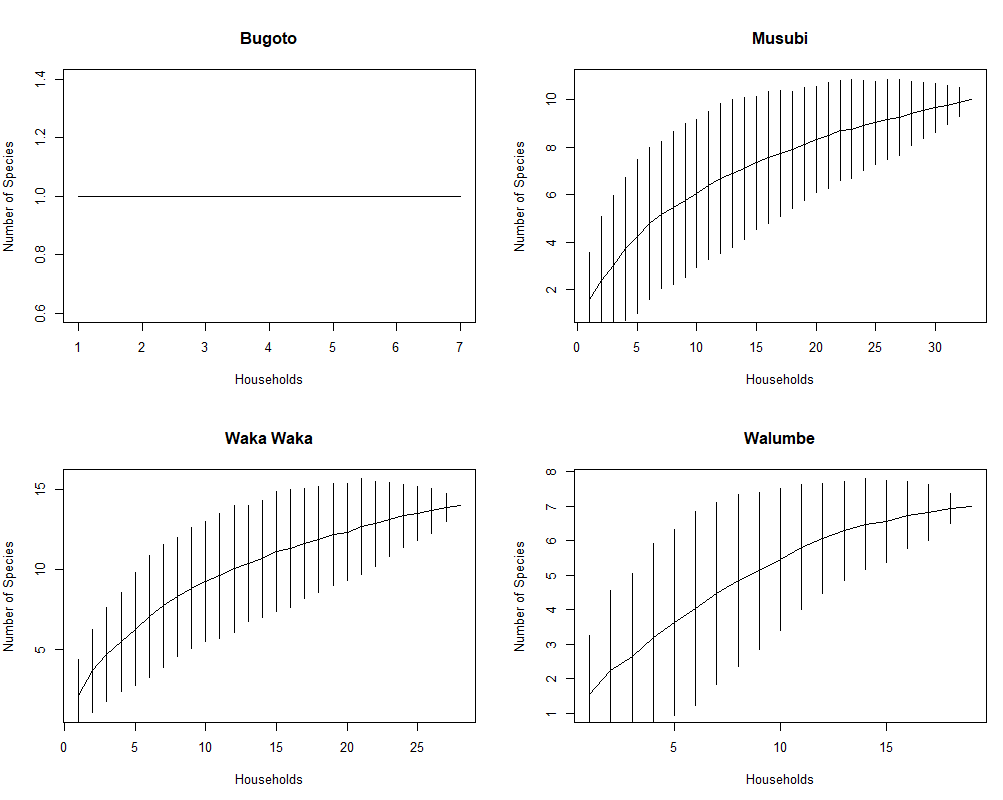


**Figure S4. A.** Species accumulation curves for all species (minimum disaggregation) and all households over two sampling periods. **B.** Species accumulation curves per village site. In Bugoto only *Rattus rattus* was trapped, resulting in a flatline curve of 1. Error bars indicate standard deviation of species richness.


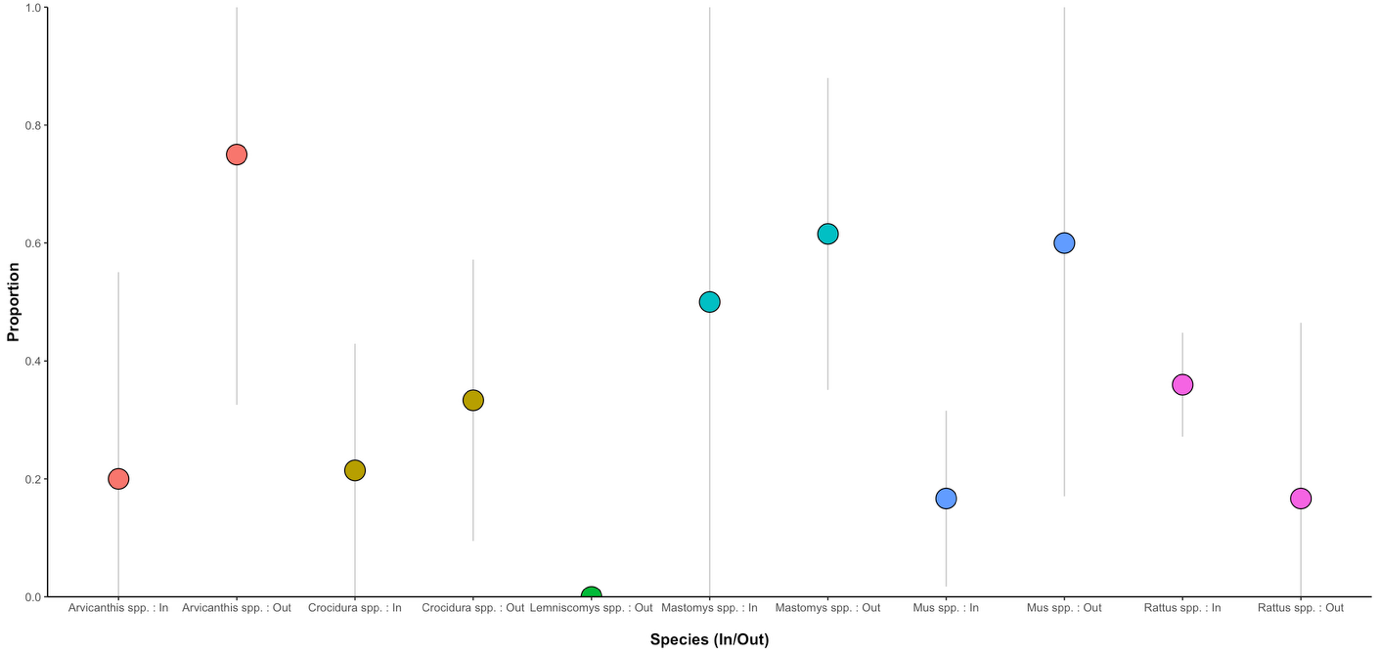


**Figure S5.** Proportion of small mammals infected with *C. hepaticum* (according to faint signal on both diluted and undiluted DNA PCR or strong signal on one diagnostic) by genus and trapping location inside or outside households. Colour coded by species. Bars indicate 95% confidence intervals.

**Table S1:** Mean and distribution (SD) of environmental characteristics per village centroid within 0.5km buffer radii

|  | | | Bugoto | Musubi | Waka Waka | Walumbe | Mean | SD |
| --- | --- | --- | --- | --- | --- | --- | --- | --- |
| Temp. scale | | |  |  |  |  |  |  |
| Elevation (m) |  | 2000 | 1140.32 | 1145.84 | 1141.15 | 1141.62 | 1142.23 | 2.47 |
| Temp. min (Feb) (°C) | 1970-2000 | | 16.50 | 16.50 | 16.50 | 16.30 | 16.45 | 0.10 |
| Temp. min (Aug) (°C) | 1970-2000 | | 15.90 | 15.90 | 15.80 | 15.80 | 15.85 | 0.56 |
| Temp. max (Feb) (°C) | 1970-2000 | | 29.60 | 29.60 | 29.70 | 29.30 | 29.55 | 0.18 |
| Temp. max (Aug) (°C) | 1970-2000 | | 27.20 | 27.20 | 27.11 | 27.10 | 27.15 | 0.06 |
| Population density (p/km^2^) |  | 2019 | 824.03 | 881.06 | 669.54 | 260.46 | 658.77 | 280.17 |
| Building density (count) |  | 2023 | 730 | 213 | 502 | 513 | 489.50 | 212.13 |
| Percentage cropland (%) |  | 2019 | 19.04 | 47.25 | 51.87 | 15.93 | 33.52 | 18.66 |
| Average forest height (m) |  | 2019 | 28.23 | 11.86 | 24.91 | 35.26 | 25.06 | 9.80 |
| Forest cover (count) | 50% | 2009 | 108 | 129 | 22 | 136 | 98.75 | 52.53 |
| *Classification threshold* | 40% | 2009 | 209 | 233 | 38 | 191 | 188 | 107.92 |
| *% canopy cover per pixel* | 30% | 2009 | 344 | 323 | 63 | 235 | 241.25 | 127.87 |
|  | 50% | 2014 | 108 | 129 | 18 | 136 | 97.75 | 54.48 |
|  | 40% | 2014 | 290 | 233 | 32 | 191 | 186.50 | 110.70 |
|  | 30% | 2014 | 344 | 323 | 57 | 235 | 239.75 | 130.66 |
|  | 50% | 2017 | 108 | 129 | 18 | 136 | 97.75 | 54.48 |
|  | 40% | 2017 | 290 | 233 | 32 | 191 | 186.50 | 110.70 |
|  | 30% | 2017 | 344 | 323 | 57 | 235 | 239.75 | 130.66 |
|  | 50% | 2019 | 108 | 129 | 18 | 136 | 97.75 | 54.48 |
|  | 40% | 2019 | 290 | 230 | 32 | 191 | 185.75 | 110.29 |
|  | 30% | 2019 | 344 | 318 | 57 | 235 | 238.5 | 129.52 |
| Forest cover (proportion) | 50% | 2009 | 0.1060 | 0.1265 | 0.0216 | 0.1337 | 0.10 | 0.05 |
| *Classification threshold* | 40% | 2009 | 0.2846 | 0.2284 | 0.0373 | 0.1878 | 0.18 | 0.11 |
| *% canopy cover per pixel* | 30% | 2009 | 0.3376 | 0.3167 | 0.0619 | 0.2311 | 0.24 | 0.13 |
|  | 50% | 2014 | 0.1060 | 0.1265 | 0.0177 | 0.1337 | 0.10 | 0.05 |
|  | 40% | 2014 | 0.2846 | 0.2284 | 0.0314 | 0.1878 | 0.18 | 0.11 |
|  | 30% | 2014 | 0.3376 | 0.3167 | 0.0560 | 0.2311 | 0.24 | 0.13 |
|  | 50% | 2017 | 0.1060 | 0.1265 | 0.0177 | 0.1337 | 0.10 | 0.05 |
|  | 40% | 2017 | 0.2846 | 0.2284 | 0.0314 | 0.1878 | 0.18 | 0.11 |
|  | 30% | 2017 | 0.3376 | 0.3167 | 0.0560 | 0.2311 | 0.24 | 0.13 |
|  | 50% | 2019 | 0.1060 | 0.1265 | 0.0177 | 0.1337 | 0.10 | 0.05 |
|  | 40% | 2019 | 0.2846 | 0.2255 | 0.0314 | 0.1878 | 0.18 | 0.11 |
|  | 30% | 2019 | 0.3376 | 0.3118 | 0.0560 | 0.2311 | 0.23 | 0.13 |

**Table S2:** Summary of results from household rodent/small mammal survey (**N=81**)

|  |  |  | **N (%)** |
| --- | --- | --- | --- |
| **Rodents sighted** | Every day |  | 58 (71.6) |
|  | More than once a week |  | 4 (4.9) |
|  | Less than once a week |  | 16 (19.8) |
|  | NA |  | 3 (3.7) |
| **Rodent evidence in house** | Every day |  | 57 (70.4) |
|  | More than once a week |  | 7 (8.6) |
|  | Less than once a week |  | 15 (18.5) |
|  | NA |  | 2 (2.5) |
| **Rodent evidence in surroundings** | Every day |  | 40 (49.4) |
|  | More than once a week |  | 9 (11.1) |
|  | Less than once a week |  | 24 (29.6) |
|  | Never |  | 6 (7.4) |
|  | NA |  | 2 (2.5) |
| **Rodent evidence in garden** | Every day |  | 12 (14.8) |
|  | More than once a week |  | 26 (32.1) |
|  | Less than once a week |  | 21 (25.9) |
|  | Never |  | 6 (7.4) |
|  | No fields |  | 13 (16.0) |
|  | NA |  | 3 (3.7) |
| **Rodent evidence in kitchen** | Every day |  | 49 (60.5) |
|  | More than once a week |  | 10 (12.3) |
|  | Less than once a week |  | 19 (23.5) |
|  | Never |  | 1 (1.2) |
|  | NA |  | 2 (2.5) |
| **Garden location** | Adjacent/less than 5 min walk |  | 30 (37.0) |
|  | More than 5 min walk |  | 37 (45.7) |
|  | NA |  | 14 (17.3) |
| **Pest control** | Yes |  | 65 (80.2) |
|  | No |  | 14 (17.3) |
|  | NA |  | 2 (2.5) |
| **Pest control type** | Chemical |  | 61 (75.3) |
|  | Mechanical |  | 4 (4.9) |
|  | NA |  | 16 (19.6 |
| **Total** |  |  | **81 (100%)** |

**Table S3.** Bivariable binomial generalised linear regression analysis of effect of landscape and ecological covariates on village level prevalence of *C. hepatica.*

|  | **Coefficient** | **CI 95%** | | **P value ^†^** |  |
| --- | --- | --- | --- | --- | --- |
| Elevation (m) | 1.127 | 0.99 | 1.28 | 0.0739 | . |
| Temperature min (°C) | 0.853 | 0.77 | 0.935 | 0.000911 | *** |
| Temperature max (°C) | 0.954 | 0.93 | 0.978 | 0.000311 | *** |
| Population density (p/km^2^) | 1.00 | 1.00 | 1.00 | 0.03802 | * |
| Building density (count) | 1.00 | 1.00 | 1.00 | 0.245 | - |
| Percentage of cropland (%) | 0.78 | 0.11 | 5.95 | 0.807 |  |
| Average forest height (m) | 0.97 | 0.94 | 1.00 | 0.068 | . |
| Forest cover (count) * | 1.00 | 1.00 | 1.01 | 0.0109 | * |
| Shannon Index (H) | 0.81 | 0.43 | 1.53 | 0.521 |  |
| Proportion of houses with rodents (HHR) (%) | 0.99 | 0.97 | 1.00 | 0.123 | - |
| Ratio (Other: *Rattus rattus*) | 0.40 | 0.22 | 0.67 | 0.000961 | *** |
| Adjusted trap success (ATS) (%) | 0.99 | 0.94 | 1.03 | 0.567 |  |
|  |  |  |  |  |  |
| Signif. codes: 0 ‘***’ 0.001 ‘**’ 0.01 ‘*’ 0.05 ‘.’ 0.1 ‘ ’ 1 | |  |  |  |  |
| * Threshold for forest = 30% tree canopy cover |  |  |  |  |  |
| ^†^ Derived from likelihood ratio test (LRT) |  |  |  |  |  |

**Table S4.** Multivariable generalized linear mixed model results for individual-level probability of infection with *C. hepaticum* (marginal R² = 0.19; conditional R² = 0.33).

|  | **aOR** | **CI 95%** | | **P value ^†^** | |  |
| --- | --- | --- | --- | --- | --- | --- |
| Ratio (Other: *Rattus rattus*) | 0.47 | 0.27 | 0.78 | 0.003837 ** | |  |
| Forest cover (count) * | 1.70 | 0.47 | 8.50 | 0.3718 | |  |
| Proportion of houses with rodents (%) | 0.69 | 0.82 | 4.70 | 0.1167 | |  |
| Signif. codes: 0 ‘***’ 0.001 ‘**’ 0.01 ‘*’ 0.05 ‘.’ 0.1 ‘ ’ 1 | | | | | | |
| * Threshold for forest = 30% tree canopy cover | | | | |  | |
| ^†^ Derived from likelihood ratio test (LRT) | | | | |  | |

**Table S5.** BLASTn results of the *C. hepaticum* partial mitogenome against NCBI’s nucleotide database. Matches listed are the top 15 hits.

| Scientific Name | Accession | Accession Length | Total Score | Query Cover | E value | Percent identity |
| --- | --- | --- | --- | --- | --- | --- |
| *Pseudocapillaria tomentosa* | MZ708958.1 | 14062 | 7068 | 89% | 0 | 78.09 |
| *Aonchotheca putorii* | NC_071371.1 | 14168 | 8762 | 97% | 0 | 79.08 |
| *Capillaria* sp. cat-2018 | MH665363.1 | 13624 | 3451 | 63% | 0 | 73.93 |
| *Eucoleus annulatus* | NC_056391.1 | 14118 | 2301 | 50% | 0 | 73.76 |
| *Trichuris arvicolae* | MZ229684.1 | 14084 | 900 | 20% | 0 | 73.33 |
| *Trichuris muris* | NC_028621.1 | 14105 | 850 | 21% | 0 | 72.78 |
| *Trichuris rhinopiptheroxella* | PQ247221.1 | 14074 | 1017 | 13% | 0 | 76.41 |
| *Aonchotheca putorii* | OP363931.1 | 652 | 640 | 5% | 3.00E-177 | 84.36 |
| *Trichuris* sp. ETH232 | MZ229688.1 | 14100 | 584 | 14% | 1.00E-160 | 72.82 |
| *Trichuris* sp. KE396 | MZ229689.1 | 14097 | 580 | 14% | 2.00E-159 | 72.75 |
| *Trichuris mastomysi* | MZ229690.1 | 14091 | 562 | 14% | 6.00E-154 | 72.66 |
| *Trichuris suis* | KT449822.1 | 14521 | 555 | 14% | 1.00E-151 | 72.71 |
| *Trichuris muris* | AP017703.1 | 14297 | 549 | 14% | 5.00E-150 | 72.66 |
| *Calodium hepaticum* | MF962896.1 | 407 | 547 | 3% | 2.00E-149 | 91.27 |
| *Trichuris* sp. ETH392 | MZ229686.1 | 14098 | 534 | 14% | 1.00E-145 | 72.4 |
